# Hyperprogression After Immune-Checkpoint Inhibitor Treatment: Characteristics and Hypotheses

**DOI:** 10.3389/fonc.2020.00515

**Published:** 2020-04-29

**Authors:** Hongjing Zang, Jinwu Peng, Hongmei Zheng, Songqing Fan

**Affiliations:** ^1^Department of Pathology, The Second Xiangya Hospital, Central South University, Changsha, China; ^2^Department of Pathology, Xiangya Basic Medical School, Central South University, Changsha, China

**Keywords:** ICIs, immunotherapy, HPD, PD-1, PD-L1, CTLA-4

## Abstract

Immunotherapies in tumors have attracted increasing attention. They play an important role in precision medicine. Many immune-checkpoint inhibitors (ICIs) have obtained FDA approval and show good performance in the clinic. Hyperprogressive disease (HPD) after ICIs was first described in November 2016. Since then, a series of cases of HPD after ICIs have been reported. Notwithstanding that only a small subset of patients may experience this atypical response, HPD in affected patients means shorter survival times and worse prognoses. We summarized common standards for HPD diagnosis and profiled advantages and disadvantages. Elderly age, *MDM2* family amplification, infiltration of PD-1-positive regulatory effector T cells and M2-like macrophages, and cancer stem cells may take part in HPD occurrence. Overall, we should focus on investigating the early markers and pathogenic mechanisms of HPD to solve this issue in ICIs.

## Background

**Figure 1:**
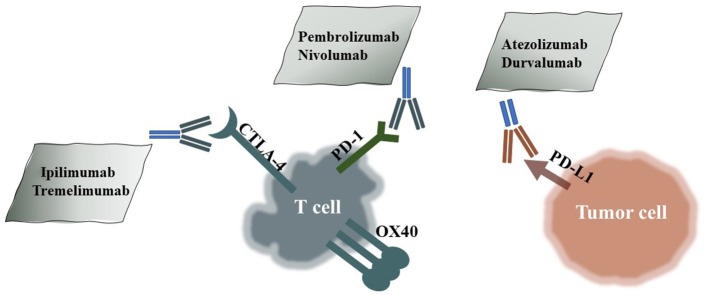
HPD-Related ICIs and Their Targets. Pembrolizumab and nivolumab are PD-1 antibodies; atezolizumab and durvalumab are PD-L1 antibodies; and ipilimumab and tremelimumab are CTLA-4 antibodies.

Immune-checkpoint inhibitors (ICIs) improve current therapies in many malignant cancers, such as non-small-cell lung cancer (NSCLC) (1), head and neck squamous cell carcinoma (HNSCC) (2), bladder cancer (3), breast cancer (4), endometrial stromal sarcoma, and renal cell carcinoma (RCC). ICIs include not only monoclonal antibodies targeting PD-1/PD-L1 and CTLA-4 (5), but also T cell immunoglobulin mucin 3 (TIM3) antibodies (6) and B and T lymphocyte attenuator (BTLA) antibodies (7). Although burgeoning targeted treatments, such as EGFR TKIs, prolong overall survival (OS) (8), the emergence of rapid drug resistance profoundly limits the long-term benefits for patients. In contrast, ICIs represent a unique and promising treatment option and complement targeted therapies in certain tumor types. Most tumor cells escape from the host immune system to protect themselves from killing by T cells, while ICIs aim to break the balance in the tumor environment and activate the immune system. Some clinical trials show significantly better OS with specific ICIs. ICIs including anti-PD-1 mAbs (pembrolizumab and nivolumab), anti-PD-L1 mAbs (atezolizumab and durvalumab), and anti-CTLA4 mAbs (ipilimumab) are approved by the FDA (Figure 1). However, unconventional responses occur in some subsets of patients after ICI treatment, such as pseudoprogression and hyperprogressive disease (HPD) (9). Both of these disorders present tumor growth on radiology scans, but the former is followed by a sharp decrease in tumor growth, while the latter is a genuine progression of the tumor. Importantly, HPD is a real phenomenon closely related to ICI utilization and is different from normal tumor progression (10).

Response Evaluation Criteria in Solid Tumors (RECIST) and RECIST 1.1 were introduced to evaluate antitumor treatment effects (11, 12). RECIST and RECIST 1.1 classify therapeutic effects into four aspects: complete response (CR), partial response (PR), stable disease (SD), and progressive disease (PD). It is obvious that PD encompasses the hyperprogression phenomenon, but it cannot distinguish HPD, pseudoprogression, and normal tumor pression with complete drug resistance. Immune-related RECIST (irRECIST) improved RECIST, and it could capture novel response patterns in immunotherapy. irRECIST improved RECIST because it could capture novel response patterns in immunotherapy (13). However, irRECIST is not suitable for HPD estimation because it does not discuss HPD, in which case HPD may be simply characterized as PD according to irRECIST. Despite the limitations, the following methods to define HPD are based on the above-mentioned criteria.

In this paper, we summarized different criteria for HPD diagnosis in solid tumors and discussed possible mechanisms and predictors of HPD.

## Appearance, Definition, and Diagnosis of Hyperprogression

HPD after ICIs was first identified by S. Chubachi (14). A 54-year-old man with recurrent NSCLC received 10th-line chemotherapy with nivolumab. 6 weeks later, obviously larger tumor lesions and newly formed lymphatic and brain metastases indicated “tumor flares” (14). It is inaccurate to define tumor “flare-up” as HPD since there is a subset of effective responses with temporary disease growth. A label of PD based on the three generations of RECIST cannot distinguish whether tumor progression is “normal” or a “flare-up.” Another atypical response type is pseudoprogression, which was first reported in melanoma after pembrolizumab treatment (15). HPD and pseudoprogression may be confused in the early stage. Pseudoprogression, in contrast to HPD, indicates good therapeutic efficacy. Chances are worse for patients whose HPD is not found or is mistaken as pseudoprogression until after it has caused severe illness. Researchers have demonstrated that HPD truly exists (10); the next step is to consider HPD as its own entity and appropriately define it.

Champiat et al. first defined “tumor flares” after immunotherapy as HPD based on RECIST 1.1 (11, 16). They used the tumor growth rate (TGR) before PD-1/PD-L1 treatments as a baseline, and an at-least 2-fold enhancement in the TGR after ICI treatments was ruled as HPD (16). TGK_R_ is defined as the ratio of the rate of tumor growth on ICI treatment to that before ICI treatment (17). Saâda-Bouzid et al. found that 29% of HNSCC patients had HPD, based on the criterion TGK_R_ ≥ 2 (17). Similar to TGR, TGK_R_ = $\left(\frac{\frac{S_{post}-S_{0}}{T_{post}-T_{0}}}{\frac{S_{0}-S_{pre}}{T_{0}-T_{pre}}}\right)$, where T is the timepoint and S is the diameter of the tumor. T_pre_, T_0_, and T_post_ mean the timepoints of the preceding baseline, baseline, and after the baseline, respectively. However, TGR is simpler and more convenient than TGK_R_, and TGR_R_ is the ratio of TGR. 1 month before the definition of TGR_R_, Kato et al. showed six cases of HPD in different solid tumors (18). They defined HPD with three criteria: (1) time to treatment failure (TTF) <2 months, (2) 50% increase in tumor burden, and (3) >2-fold increase in progression rate (18). Tumor burden is estimated by RECIST 1.1, and tumor progression rate is estimated by irRECIST. Kato's definition benefits the early discovery of HPD more than the TGR or TGK definitions and takes new lesions into consideration.

Another group believed that these definitions did not consider patient clinical status, so they introduced Eastern Cooperative Oncology Group (ECOG) to evaluate patient performance as one criterion (19) (Table 1). Taking these into consideration is a double-edged sword, because it may mistake PD for HPD.

**Table 1:** Different criteria for HPD.

**Name**	**Cancer types**	**Applications**	**Definition**	**Criteria**	**Advantages**	**Disadvantages**	**Reference (year)**
RECIST	Solid tumors	Tumor therapeutics	PD	≥20% increase in size	More accurate assessments for treatment response than before	HPD undefined	(12) (2001)
RECIST 1.1	Solid tumors	Tumor therapeutics	PD	≥20% increase in the sum of diameters of target lesions (new lesions are also considered progression)	Improvement in dimension assessments; newer imaging technologies; new lesions are considered	HPD undefined	(11) (2009)
irRECIST	Solid tumors	Antitumor immunotherapy	irPD	≥25% increase in tumor burden, repeatable	Specific for immunotherapy	HPD undefined	(13) (2009)
TGR_R_	Solid tumors	PD-1/PD-L1 inhibitors	HPD	TGR_R_ ≥2	First introduced HPD definition	Pre-ICI treatments details are necessary; reference period is limited	(16) (2017)
TGK_R_	R/M HNSCC	PD-1/PD-L1 inhibitors	HPD	TGK_R_ ≥2	Pseudoprogression and HPD can be distinguished; simpler calculation	Pre-ICI treatments details are necessary	(17) (2017)
Kato et al. criteria	Multiple types of solid tumors	Immunotherapy agents	HPD	TTF <2 months; 50% increase in tumor burden; >2-fold change in progression rate	Less time for HPD recognition	Clinical status changes are ignored	(18) (2017)
Lo Russo et al. criteria	Multiple types of solid tumors	ICIs	HPD, ≥3 criteria	TTF <2 months; 50% increase in tumor lesions; ≥ 2 new lesions; spread of disease; clinical deterioration by ECOG	Applicable for first-line treatment with ICIs	Higher false positive	(19) (2019)

*PD, progressive disease; R/M HNSCC, recurrent/metastatic head and neck squamous cell carcinoma; TGK_R_, ratio of the rate of tumor growth on ICI treatment to that before ICI treatment*.

The evolution and development of these standards are summarized in Table 1.

## Tumor Progression Under ICI Treatment

The majority of HPD cases occurred during anti-PD-1/PD-L1 treatment, and a minority occurred during CTLA-4 treatment.

### PD-1/PD-L1

Pseudoprogression was first reported in CTLA-4 therapy in advanced melanoma (24 of 327 patients; 7.3%) (15). It is characterized by tumor depression after rapid progression. The biopsy results show lymphocyte infiltration and tumor necrosis. In fact, pseudoprogression indicates favorable effects of ICI treatments.

Another atypical type of response after ICIs, tumor “flare-up,” was first reported in NSCLC after treatment with nivolumab, a PD-1 inhibitor (14). The phenomenon occurred in a 54-year-old man after a series of treatments: irradiation therapy, EGFR TKIs, cytotoxic agents, and nivolumab. After nivolumab, according to imaging detection, his tumor progressed rapidly, and new brain metastases were observed (14). Nivolumab is one of the current FDA-approved PD-1 antibodies, and the other is pembrolizumab; PD-L1 antibodies compromise atezolizumab and durvalumab. Champiat et al. defined HPD for the first time (16). They collected 131 eligible patients with multiple types of solid tumors, and 7 out of 78 (9.0%) patients treated with PD-1 inhibitors and 5 out of 53 (9.4%) patients treated with PD-L1 inhibitors developed HPD (16). According to their research, there was no significant difference in the HPD occurrence rate between anti-PD-1 and anti-PD-L1 therapies (*p* = 1) (16).

Saâda-Bouzid et al. collected HNSCC patients who were not covered by a previous study (17). 10 of 34 patients (29.4%) were diagnosed with HPD, and the difference in the HPD occurrence rate between anti-PD-1 and anti-PD-L1 treatments in recurrent and/or metastatic HNSCC patients was also not statistically significant (*p* = 0.23) (17). As expected, HPD predicts a worse prognosis: decreased progression-free survival (PFS) and OS (17). Another manuscript investigated HPD in digestive system malignancies (20). Among 25 patients, 5 were diagnosed with HPD, 4 of whom received the PD-L1 inhibitor atezolizumab, while the rest received CTLA-4 and PD-L1 inhibitor combination treatment, which will be discussed later (20).

Kato et al. found 6 patients with *MDM2/4* amplification in 155 patients, and they were all diagnosed with HPD after immunotherapies. 5 of 6 patients received anti-PD-1/PD-L1 therapies (18). With the same criteria for HPD, another study diagnosed 4 of 36 advanced gastric cancer patients treated with nivolumab as having HPD (21). A large experiment with 406 eligible advanced NSCLC patients proved that HPD is more common with anti-PD-1/PD-L1 therapies than with chemotherapies (22). A case of HPD in melanoma was also reported in a 25-year-old female after combination therapy with ipilimumab, nivolumab plus trametinib, and dabrafenib (23). Another study reported that two metastatic urothelial carcinoma patients were diagnosed with HPD after anti-PD-1 mAb treatment and died soon after (24). Intriguingly, rapid tumor progression after PD-1 inhibitor treatment has also occurred in leukemia (25). The HPD occurrence rate seems not to be significantly different between anti-PD-1 mAbs and anti-PD-L1 mAbs (22).

In conclusion, a subset of patients may suffer worse prognosis from PD-1/PD-L1 inhibitors than from other therapy types, and HPD may not be related to specific PD-1 or PD-L1 antibodies. The characteristics of these cases are summarized in Table 2.

**Table 2:** Characteristics of HPD Cases.

**Drug**	**HPD cases**	Occurrence*	**Average age**	**Sex (male/female)**	**Gene mutations**	**Cancer histology**	**Reference**
Anti-PD-1 or PD-L1 mAbs*	22	9–23%	≥63	12/10	/	Melanoma, colorectal cancer, urothelial cancer, ovarian cancer, cholangial cancer, lung cancer	(16, 17)
Anti-PD-1 mAbs	14	~11%	65.7	9/5	*MDM2* amplification; *KIF5B-RET* fusion; *CDK4* amplification; *ERBB2* amplification, *KRAS* amplification,	Gastric cancer, breast cancer, endometrial cancer, lung cancer, liver cancer, bladder cancer	(18, 21), (24, 26)
Anti-PD-L1 mAbs	5	~18%	59	3/2	*MDM2* amplification, HER-2 positivity	Bladder cancer, gastric cancer, colorectal cancer, esophageal cancer	(18, 20)
Anti-PD-L1 mAbs + CTLA inhibitor	2	~4%	59	2/0	/	Esophageal cancer, liver cancer	(20, 26)
Anti-PD-1 mAbs + CTLA inhibitor	1	/	25	0/1	*BRAF V600E* mutation	Melanoma	(23)
OX40 agonist	1	/	62	1/0	*MDM4* amplification	Hypopharynx cancer	(18)

/, unmentioned in the reference; *, Specific single drug cannot be distinguished by the reference.

### CTLA-4 Antibodies

Cytotoxic T lymphocyte-associated protein 4 (CTLA-4), also named CD152, suppresses antigen-presenting cells. Similar to PD-L1/PD-1, interaction with CTLA-4 attenuates T cells and leads to immunosuppression (26). The only FDA-approved CTLA-4 inhibitor is ipilimumab (27). Another CTLA-4 antibody, tremelimumab, is still in clinical trials. Zhi et al. reported that a 49-year-old man with esophageal neuroendocrine carcinoma (NEC) showed new spinal, liver, and lung metastases after 6 weeks of durvalumab and tremelimumab combination treatment (20). In another study, 3 of 19 patients treated with a CTLA-4 inhibitor alone and 2 of 16 patients treated with combination treatment with a CTLA-4 inhibitor and anti-PD-1 had a TTF <2 months (18). However, the authors did not further verify whether these cases were HPD or not (18). Another study found that one patient treated with a single-agent CTLA inhibitor and one patient treated with combination treatment with a CTLA-4 inhibitor and a PD-L1 inhibitor were characterized as having HPD (28).

Moreover, HPD has rarely been seen in patients treated with a single-agent CTLA-4 inhibitor. Overall, CTLA-4 inhibitors are not the main treatments responsible for HPD.

### OX40 Agonist-Related

OX40 (CD134), which is highly expressed by T cells, belongs to the TNF receptor family. In fact, OX40 agonists are not strictly ICIs. They aim to activate OX40 rather than inhibit it (29). The combination therapy including an OX40 agonist and PD-L1 blockade is recommended in research (30). However, a 62-year-old man was diagnosed with HPD after OX40 agonist therapy (18). This patient, who had a hypopharyngeal squamous cell tumor, harbored MDM4 amplification and died 4.4 months after OX40 agonist initiation (18). No other OX40 agonist HPD cases have been reported. Nevertheless, we should not neglect the possibility of HPD occurrence in the clinic (Table 2).

## Possible Mechanism and Predictors for HPD

If we treat HPD as a special type of drug resistance, according to common classification terms, is HPD an intrinsic resistance, an acquired resistance, or both?

### Intrinsic Resistance?

Kamada et al. hypothesized that PD-1-positive regulatory T cells (Tregs) play key roles in anti-PD-1-mediated HPD in advanced gastric cancer (21). They found that in non-HPD patients, the ratio of eTregs:CD8^+^ cells, the ratio of Ki67^+^ Tregs:Ki67^+^CD8^+^ cells, and the percentage of Ki67^+^ Tregs decreased significantly after nivolumab treatment, while they remained stable or even slightly increased in HPD patients (21). Interestingly, CTLA-4 is highly expressed in effector Tregs (31). CTLA-4 treatments increased Ki67^+^ Tregs (21). Anti-CTLA-4 mAb and anti-PD-1 mAb combinations were associated with less HPD occurrence in the clinic than other ICI combinations (20).

Another group found 39 HPD patients among 187 NSCLC patients, and M2-like macrophage (CD163^+^CD33^+^PD-L1^+^) infiltration in tumors was found in all HPD patients (19). In their animal study, nivolumab-related HPD showed infiltration of M2-like macrophages, which was thought to be caused by Fc (of nivolumab)-Fcγ receptor binding.

**Figure 2:**
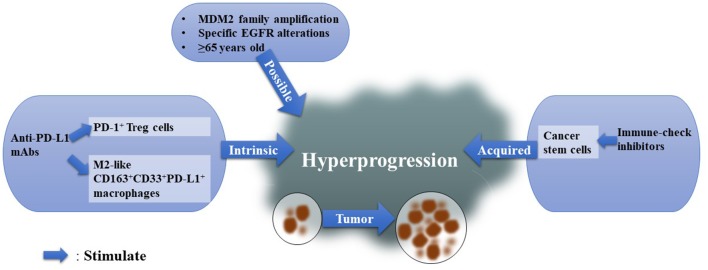
Possible Mechanisms for HPD. We summarized existing mechanisms for HPD and classified them into three types: intrinsic immunological reasons, acquired elements, and possible factors.

If PD-1+ Tregs and M2 macrophage infiltration induced by nivolumab are the major mechanisms responsible for HPD, it is difficult to explain why there is no significant difference between anti-PD-1 mAbs and anti-PD-L1 mAbs. The sample size for anti-PD-L1-related HPD may be too small. Or do anti-PD-L1 mAbs also stimulate infiltration of PD-1^+^ Tregs and M2-like macrophages? In fact, anti-PD-1 and anti-PD-L1 mAbs are two different types of immunoglobulin G (IgG). The former belongs to the IgG4 class, which inactivates the relevant pathway by binding with the inhibitory receptor, FcγRIIb, and the latter belongs to the IgG1 family, leading to cell death by binding with activating receptors: FcγRI, FcγRIIa, and FcγRIIIa. This means that the Fc–Fcγ interactions are very different (32) (Figure 2).

### Acquired Resistance?

Cancer stem cells (CSCs) may contribute to the acquired resistance hypothesis of HPD. Cytotoxic T lymphocytes (CTLs), the cells that respond when ICIs stimulate the host immune system, were found to favor cancer cell stemness if cancer cells survived (33). Notably, PD-L1 was found to maintain the stem-like phenotype of breast cancer cells (34) (Figure 2).

### Coincidence or Significance?

In the study of Kamada et al. only 1 among 31 patients had *MDM2* amplification, and this patient suffered HPD (21). This case supports the idea that HPD after a single PD-1/PD-L1 inhibitor may be more frequent in patients with *MDM2* family amplification than in patients without *MDM2* amplification (18). Inhibiting the PD-1 pathway could induce an increase in Interferon-γ (IFN-γ) (35), while IFN-γ can stimulate the JAK-STAT pathway (36), and IFN regulator factor-8 (IRF-8), a downstream factor of JAK-STAT (37), may induce *MDM2* overexpression (38, 39). This hypothesis, raised by Kato et al. explains why HPD is more frequent in patients with MDM2 family amplification. However, further verification is needed *in vivo* and *in vitro*. They also found *EGFR* alterations in HPD patients. Another study compared somatic mutations in two HPD patients before and after anti-PD-1 therapies and found that the two HPD patients harbored both more mutations after ICIs and significantly decreased immune scores (40). Notably, enriched ILC3 marker genes after anti-PD-1 treatments indicate that ILC3s may participate in HPD (40). Age is also an important factor, as HPD is more common in elderly patients (age ≥ 65) (16, 22, 41) (Figure 2).

In conclusion, *MDM2* family amplification and older age (≥65) are possible risk factors for HPD. Even though PD-1^+^ Tregs, M2-like macrophage infiltration and ICI-stimulated CSCs have been presented as possible hypotheses for the HPD mechanism, there is still an urgent need to understand the occurrence of HPD and identify predictive factors for early diagnosis.

## Conclusion

With increased awareness of tumors, treatment methods have improved from broad approaches (surgery and cytotoxic agents) to precision medicine (targeted treatments). ICIs are promising. However, HPD intimidates doctors and patients. Once HPD occurs, ICIs are not only invalid for tumor treatment but also detrimental for patients. HPD always indicates poor OS, increased metastasis and rapid tumor growth.

Currently, there are three different criteria for HPD diagnosis: (1) the TGR_R_ criteria (16); (2) the TGK_R_ criteria (17); and (3) the Kato et al. criteria (18). They are all widely used in research. Another set of criteria takes clinical status into consideration (19) (Table 2).

The mechanism of HPD and methods to predict it remain unclear. Recognition of HPD always occurs after rapid tumor growth, which may be too late for patients. The sooner the ominous progression is identified, the quicker we can stop ICIs to rescue this small subset of patients. *MDM2* amplification, specific *EGFR* mutations and older age may contribute to HPD. PD-1-positive Tregs and M2-like macrophages play important roles in HPD caused by PD-1 inhibitors, with obvious limitations and outstanding questions. There is still a need for research with larger sample sizes and deeper investigations.

At the present stage, distinct therapies such as EGFR TKIs, ICIs, cytotoxic agents, and radiotherapy should not be used in isolation. Various combinations are worth trying in animal studies. Even within ICIs, different combinations should be investigated to explore ways to increase efficacy and lessen severe side effects. Since HPD occurrence in anti-PD-L1 mAbs is around 18%, while it's around 4% in anti-PD-L1 mAbs combined with CTLA inhibitor (Table 2), we recommend combination therapy for patients with risk factors (for example: elder age and *MDM2* amplification).

In conclusion, it is urgent to identify specific predictive markers that could predict HPD early after ICI treatment and to develop effective methods to prevent HPD, which requires further insight into the mechanisms of HPD.

## Author Contributions

SF: substantial contributions to the conception and design of the work, agreed to be accountable for all aspects of the work in ensuring that questions related to the accuracy or integrity of any part of the work are appropriately investigated and resolved. HZa: drafting the work and revising it critically for the whole manuscript. JP: responsible for mechanism part, instruct to improve this part, and provide related reference. HZh: responsible for manuscript structure and English grammar. All authors: contributed to manuscript revision, read, and approved the submitted version.

## Conflict of Interest

The authors declare that the research was conducted in the absence of any commercial or financial relationships that could be construed as a potential conflict of interest.
